# IS THERE CORRELATION BETWEEN HUMAN PAPILLOMAVIRUS (HPV) AND ESOPHAGEAL EPIDERMOID CARCINOMA?

**DOI:** 10.1590/0102-672020200002e1528

**Published:** 2021-05-14

**Authors:** Luiz Filipe Alkamin WOELLNER, Juliano Smaniotto de MEDEIROS, Carmen Australia Paredes Marcondes RIBAS, Paulo Afonso Nunes NASSIF, Jurandir Marcondes RIBAS-FILHO, Ana Cristina Lira SOBRAL, Bruno Luiz ARIEDE, Debora Azeredo Pacheco Dias DA COSTA, Osvaldo MALAFAIA

**Affiliations:** 1 Postgraduate Program in Principles of Surgery, Mackenzie Evangelical Faculty of Paraná, Curitiba, PR, Brazil; 2 Institute of Medical Research, Faculdade Evangélica Mackenzie do Paraná, Curitiba, PR, Brazil; 3 Evangelical University Mackenzie Hospital, Curitiba, PR, Brazil

**Keywords:** Carcinoma, Squamous Cell, Esophagus, Papillomaviridae, Genes, p16., Carcinoma de células escamosas, Esôfago, Papillomaviridae, Genes p16

## Abstract

***Background*:**

Currently, persistent human papillomavirus (HPV) infection has been related in some geographic regions as a risk factor for esophageal squamous cell carcinoma. It results in the immunoexpression of the p16 protein, which has been used as marker of the oncogenic lineage by this etiological agent.

***Aim*:**

To correlate epidemiological aspects of esophageal squamous cell carcinoma with the prevalence of HPV infection.

***Methods*:**

Fifty-eight cases were analyzed and submitted to histopathological and immunohistochemical analysis by p16.

***Results*:**

Of the 58 cases evaluated, 40 were men and 18 women, with a mean age of 63.2 years. p16 immunoexpression was positive in 46.55%.

***Conclusion*:**

The prevalence of HPV infection is high in esophageal squamous cell carcinoma presenting in almost half of the cases (46.55%), without gender differentiation.

## RESUMO

**Racional:**

Atualmente a infecção persistente pelo papilomavírus humano (HPV) tem sido relacionada em algumas regiões geográficas como fator de risco para o carcinoma epidermoide do esôfago. Ela resulta na imunoexpressão da proteína p16, que tem sido utilizada como marcadora da linhagem oncogênica por este agente etiológico.

**Objetivo:**

Correlacionar aspectos epidemiológicos do carcinoma epidermoide do esôfago com a prevalência de infecção pelo HPV.

**Métodos:**

Foram analisados 58 casos buscando-se perfil epidemiológico dos pacientes, com suas peças submetidas à análise histopatológica e imunoistoquímica pelo p16.

**Resultado:**

Dos 58 casos avaliados, 40 eram homens e 18 mulheres, com idade média de 63,2 anos. A imunoexpressão pelo p16 foi de 46,55%.

***Conclusão*:**

A prevalência de infecção pelo HPV é alta no carcinoma epidermoide de esôfago apresentando-se em quase a metade dos casos (46,55%), sem diferenciação de idade quanto aos gêneros.

## INTRODUCTION

Human papillomavirus (HPV) is well known and described as one of the agents that cause the development of cervical, vulvovaginal and anorectal cancers^6^ ^,^ ^8^, with subtypes 16 and 18 responsible for up to 80% of these malignancies^17^. The first reports of its involvement in squamous cell carcinoma (SCC) of the esophagus appeared in 1982 with Syrjänen^18^ ^,^ ^19^. Since then, over 100 studies have attempted to relate this oncogenic virus infection - particularly the strains 16 and 18 high risk - with this esophageal neoplasia^5^ ^,^ ^15^ ^,^ ^18^ ^,^ ^21^.

In 2013, Syrjänen^18^ made a meta-analysis based on 152 eligible articles selected from the existing literature correlating HPV detection in esophageal SCC and concluded that there is great variability of HPV in these lesions in different geographic regions. Observing the results, he also stressed that it is not because of different virus identification techniques, but because of the geographical origin of the study^7^ ^,^ ^20^. Thus, he stated that the data corroborate the newly elaborated concept that esophageal SCC may have a different cause in low- and high-risk geographic regions, with HPV playing an important role only in high-risk regions^19^.

Knowing that HPV culture is not possible, all viral tests in use are based on the detection of viral nucleic acids. These tests can be divided into indirect examination methods (such as p16 immunoexpression), signal amplification methods (such as hybrid capture) and target amplification methods (such as PCR^4^). The immunohistochemical staining for p16 was the method chosen for this work for its cost-effective, high sensitivity, low cost and simplicity^1^ ^,^ ^3^ ^,^ ^10^ ^,^ ^11^ ^,^ ^14^.

The objective was to evaluate the existence of a relationship between esophageal SCC and HPV infection in patients undergoing esophageal resection due to neoplasia. 

## METHOD

This study was carried out based on a cross-sectional and retrospective search in the electronic archives of the Evangelical University Mackenzie Hospital in Curitiba, Curitiba PR, Brazil, in search of all the cases with pathological diagnosis confirming esophageal epidermoid cancer, which occurred in the period from January 2010 to January 2017. The project was approved by the institution’s Research Ethics Committee under opinion number 1,952,103.

From the selected patients, data related to gender and age were compiled from the medical record. Paraffin blocks and histopathological slides stained by the H&E technique were obtained, both containing samples with previous diagnosis of esophageal SCC. Exclusion criteria were medical records whose reports did not include all the information relevant to the study, cases in which the slides and blocks selected by the electronic medical record were not found, as well as those that, after the assembly of the TMA (tissue microarrays or multi-sample tissue blocks) did not demonstrate neoplasia.

The histopathological slides were revised in order to select the sample areas for making multi-sample tissue blocks. After making these blocks, they were used to make the multi-sample slides, in which biomarkers were tested by immunohistochemistry to verify the presence or absence of positivity for p16.

### Immunohistochemistry technique

**FIGURE 1:**
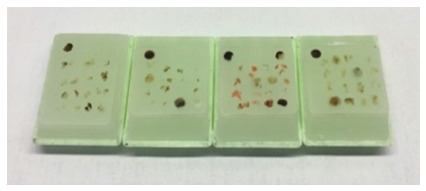
TMAs constructed for this study: Each contained eight cases with a sample of each case, in addition to a tissue sample signaling the beginning of the Cartesian map, that is, just demonstrating where the pathologist should start reading

The technique performed for the construction of TMAs or multi-sample blocks consisted of assembling paraffin blocks, with multiple samples, for making histological slides with several samples, in order to perform immunohistochemical techniques on several tissues at once, reducing material cost. From the histological slides referring to the study cases, the sample regions representative of the lesion were selected and duly marked with a retroprojection pen. Through the mirror system, the marked slide was used to locate the same region in the donor block. The technique also consisted of making a perforation in the donor block in the sample area previously marked with the consequent obtaining of a tissue cylinder inside the needle and, after that, the implantation of this tissue in the recipient block (Figure 1). Each sample fragment removed from the donor block was placed in the recipient block according to the “Cartesian plane” type map - the columns were identified with letters and the lines with numbers.

The histological slides from the handmade TMA blocks were subjected to immunohistochemistry. This process was divided into two distinct phases: the application of the standardized technique in the study material and the reading of the positive area (with positive tissue immunostaining) on ​​the TMA slides previously immunostained.

For reading, two slides of normal esophagus immunostained with p16 antibodies were used, which were used as a standard for serial definition of the mean area of ​​expression of the markers on the slides of the selected cases, using the Image Pro Plus^®^ software.

After determining the average area of ​​expression of the markers in each delimited region, the collection of data from the anatomopathological reports began.

**FIGURE 2:**
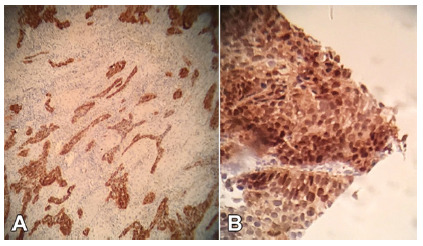
A) ECC positive for p16 (brown area x100); B) detail of positivity to p16 with strong cytoplasmic and nuclear marking (brown x400)

### Statistical analysis

All results and information obtained were tabulated according to the data protocol, and then expressed through graphs and tables, developed from Excel spreadsheets. In this study, a chi-square test was used to analyze the difference between the observed result and the expected result. However, this test is limited. When more than ¼ of the expected values ​​in the 2x2 table were less than 5, Fisher’s exact test and Cramer’s V statistic were used. For tables with variables that had more than two parameters, greater than 2x2, logistic regression was performed for analysis and dependency. The reliability was 95%.

## RESULTS

The present sample consisted of 66 cases, eight of which were excluded because the anatomopathological materials were not found, due to the fact that they did not correspond with the anatomopathological report or due to the assembly of the multi-sample slides having any technical error. Then 58 cases were selected. There were 40 men (68.97% of the total) and 18 women (31.03% of the total) with ages varying between 31-92 years in men (63.2±12.92) and in women 34-79 years (62,27±10.82, Table 1).

The measure of relative dispersion called “coefficient of variation”, used for the comparison of different distributions, for example, the differences between ages when comparing men and women, found values ​​equal to 20.32% for men and 17,39% for women, with a total of 19.36%. Thus, it can be considered that the two groups (male and female) and the general group had homogeneous ages (coefficient of variation less than 30%), with the female being more homogeneous than the male (Table 1).

**TABLE 1:** Descriptive measures regarding the age of patients

	Men	Women	General
Average	63.62	62.27	63.2
Standard deviation	12.92	10.82	12.23
Maximum	92	79	92
Minimum	31	34	31
Coefficient of variation (%)	20.32%	17.39%	19.36%
Variance	167.16	117.27	149.74

Test F p=0,43593657; t Student p=0,3508532

The prevalence of p16 positivity in this sample was 46.55% (n=27), 29.31% in men and 17.24% in women.

Regarding the distribution between age groups, there was no statistical significance (p=0.51) with the immunostaining by p16 and neither in the distribution between less and over 60 years old (p=0.7806).

## DISCUSSION

Since the relationship was first reported in 1982, attempts have been made to establish the prevalence of HPV infection in esophageal SCC^12^. The p16 protein, which acts as a tumor suppressor by competitively inhibiting cyclin-dependent kinases, is an important example of this new diagnostic lineage. It is found in low concentrations in the normal epithelium, with its expression increased in proliferative and inflammatory processes^16^. The main reason for it to be studied, allowing the analysis of viral infection in these tumor types, is the fact that, although uncertain, HPV positive patients have shown better prognosis, as well as better response to treatment with neoadjuvant chemotherapy^11^ ^,^ ^13^.

In the present study, there was a higher prevalence of esophageal SCC in men, with 40 out of 58 patients (68.97%, 2.44: 1 ratio), with a mean age of 63.2 years, which is slightly below, but it corroborates with the data released by INCA (National Cancer Institute - Brazil) in 2016, in which such index was 73.54%. In addition, this higher prevalence in men is also found in the research conducted by Kumar et al.^11^ in India, which showed a male:female ratio of 1.97:1. The average age found by Antunes et al.^2^, in a study in the city of Santa Maria, in Rio Grande do Sul, Brazil was 60.1 years, also being more prevalent in men (84.3%). Saxena et al.^17^ and Kumar et al.^11^ also reported no statistical difference between genders and positivity for HPV.

Immunoexpression of the p16 protein is a biological marker related to neoplastic induction by HPV. It showed a prevalence of 46.55% in the present series and Kumar et al.^11^ described that among 101 esophageal SCC biopsies, this expression was present in 22 cases (22%). In a meta-analysis based on 138 studies, published by Hardefeldt et al.^9^, the average prevalence was 27.4% in 12,037 cases of esophageal SCC. Comparing the underdeveloped with developed countries, emerging countries have a 12.2% higher HPV rate. Antunes et al.^2^, on the other hand, reported that the prevalence of HPV infection is not associated with SCC. In the meta-analysis of 33 studies carried out by Wang et al.^20^, 46.5% were positive cases for HPV, very close to that found in this study. Possibly the discrepancy in the values in the literature and this study, may be related to the sample size, and also to the geographical variation of the HPV carcinogenic role described by several authors.

The present study aimed to expand the understanding of the oncogenesis of esophageal SCC and the relationship of a possible risk factor. The highest limitation of this study was the sample size and also, due to the difficulty of accessing greater data related to patients; it was only possible to assess the presence or absence of HPV in the pathological process, without making correlations with other risk factors of this neoplasm.

Despite its positive results, it would be interesting to increase the number of cases in order to have a greater representation of these results in the Brazilian territory. Also, other studies should be encouraged to compare, for example, the biological behavior of these tumors with HPV with the prognosis of those without HPV.

## CONCLUSION

The prevalence of HPV infection is high in esophageal squamous cell carcinoma, presenting in almost half of the cases (46.55%), without differentiation of age as to genders.
